# Exploring the antioxidant, antidiabetic, and antibacterial potential of postbiotic compounds derived from *Lactiplantibacillus plantarum* O7S1

**DOI:** 10.5114/bta.2024.141802

**Published:** 2024-09-30

**Authors:** Nadia Aliouche, Mohamed Sifour, Houria Ouled-Haddar

**Affiliations:** Laboratory of Molecular Toxicology, Faculty of Nature and Life Sciences, University of Jijel, 18000 Jijel, Algeria

**Keywords:** *Lactiplantibacillus plantarum* O7S1, postbiotics, antioxidant activity, antibacterial activity, antidiabetic activity

## Abstract

Probiotic bacteria are distinguished by their ability to produce various functional postbiotic metabolites. Therefore, this study aimed to explore the *in vitro* antioxidant, antidiabetic, and antibacterial properties of two postbiotics generated by *Lactiplantibacillus plantarum* O7S1 (*Lpb. plantarum* O7S1) during the fermentation process: cell-free supernatant (CFS) and exopolysaccharides (EPS). The antioxidant potential of these postbiotics was assessed using various radical scavenging assays and ferric-reducing antioxidant potential. The antidiabetic activity was evaluated through α-amylase inhibitory assays, while antibacterial activity was determined using agar well diffusion assays. The results of the present study revealed that CFS exhibited significant antioxidant and antidiabetic efficacy in contrast to EPS (*P* < 0.001). Specifically, CFS displayed remarkable scavenging ability against DPPH, hydroxyl, and superoxide radicals, with inhibition rates of 88.78, 78.91, and 34.85%, respectively, while EPS showed comparatively lower inhibition rates. Additionally, CFS demonstrated higher reducing activity (0.30 optical density units at 700 nm) and potent α-amylase inhibitory activity (95.87%) compared to EPS (67.17%) (*P* < 0.001). The agar well diffusion assay reported that CFS showed significant antimicrobial activity against both Gram-positive and Gram-negative pathogens, while no activity was observed with EPS. Furthermore, carbohydrate fermentation analysis indicated the strain’s ability to metabolize various carbohydrates and their derivatives, potentially enhancing digestive health. These findings suggest that both CFS and EPS exhibit promising hypoglycemic, antioxidant, and antibacterial properties, making them potential candidates for incorporation into functional foods and pharmaceuticals aimed at preventing oxidative damage, diabetes, and pathogenic bacterial infections.

## Introduction

With increasing health awareness, consumers are now looking for new nutritional strategies to modulate gut microbiota and improve overall health. The use of beneficial microorganisms, such as probiotics, has been established as a potential tool for enhancing gut health in several reports (Rastogi et al., 2020; Ahire et al., 2021; Lorizzo et al., 2022). The International Scientific Association for Probiotics and Prebiotics (ISAPP) defines probiotics as “live microorganisms that, when administered in adequate amounts, confer a health benefit on the host” (Hill et al., 2014). Various studies have documented their beneficial effects, which include reducing lactose intolerance, preventing antibiotic-associated diarrhea, alleviating oxidative stress, inflammation response, and metabolic diseases such as diabetes, and improving gastrointestinal function by preventing the proliferation of pathogenic bacteria (Nataraj et al., 2020; Rastogi et al., 2020; Abbasi et al., 2022).

However, despite the effectiveness of probiotics in improving health, recent evidence suggests that the use of probiotic cells has some limitations, particularly concerning their viability, colonization in the gastrointestinal tract, the emergence of antibiotic resistance, and the transfer of virulence genes in the gut microbiome (Nataraj et al., 2020; Aghebati-Maleki et al., 2021). To overcome these issues, a recent emerging trend suggests that bacterial viability is not necessary to obtain the beneficial effects of probiotics (Aguilar-Toalá et al., 2018; Rafique et al., 2023). This trend has been confirmed by several reports over the past few years (Aguilar-Toalá et al., 2018; Aydin et al., 2021; Abbasi et al., 2022). Therefore, the use of inanimate forms and metabolites of probiotic bacteria (postbiotics) could be a good alternative to live probiotic cells due to their safety, longer shelf life, and stable chemical composition (Moradi et al., 2021; Rafique et al., 2023).

In general, postbiotics have demonstrated all the beneficial effects of probiotics, including antioxidant, antiobesity, anticancer, antidiabetes, hypocholesterolemic, and anti-inflammatory effects; these have been proven both *in vitro* and *in vivo* (Aguilar-Toalá et al., 2018; Aghebati-Maleki et al., 2021; Noori et al., 2023). Postbiotics are defined as any factor resulting from the metabolic activity of a probiotic or any released molecule during the fermentation process (in culture media or the gut) capable of improving health and/or well-being when consumed in sufficient quantities (Mayorgas et al., 2021; Moradi et al., 2021). Lactic acid bacteria, particularly the *Lactiplantibacillus* species, constitute the most important group of probiotics showing the ability to produce many types of postbiotics in the culture media or the gut microbiome after the catabolism of carbohydrates, proteins, or lipids due to their enzymatic systems (Diez-Gutiérrez et al., 2022).

To our knowledge and based on several studies focusing on this subject, the variability in the metabolism of *Lactiplantibacillus plantarum* strains should substantially impact other metabolic pathways involved in the production of postbiotics, which could have several beneficial effects on human health (Diez-Gutiérrez et al., 2022). Regarding the postbiotic metabolites, CFS containing organic compounds, amino acids, proteins, and EPS plays a vital role as a free-radical scavenger for the prevention of oxidative stress. Moreover, it serves as an inhibitor or reducer of carbohydrate digestion by enzymes like α-amylase and α-glucosidase, thereby helping to regulate postprandial hyperglycemia. Additionally, it demonstrates efficacy in inhibiting pathogens (Aghebati-Maleki et al., 2021; Noori et al., 2023).

Considering the abovementioned background, the present study aimed to assess the ability of *Lpb. plantarum* O7S1, isolated from fermented olives, to produce two postbiotics (CFS and EPS). Furthermore, the research aimed to explore their antioxidant, antibacterial, and antidiabetic activities. Additionally, an analysis of the carbohydrate fermentation profile of this strain was conducted to assess its potential for postbiotic production in the intestine or food products.

## Materials and methods

### Bacterial strains and growth conditions

The lactic acid bacterium used in this study was isolated from fermented olives and was selected for its probiotic traits and its capacity for EPS production at the Laboratory of Molecular Toxicology, University of Jijel, Algeria. This strain was identified genotypically by 16S rRNA gene sequencing as *Lpb. plantarum* O7S1 (GenBank Accession Number: PP575676). Additionally, seven clinical bacterial strains were employed as indicators for antimicrobial activity: *Escherichia coli* ATCC 29522, *Staphylococcus aureus* ATCC 29523, *Pseudomonas aeruginosa* ATCC 27853, methicillin-resistant *S. aureus* (MRSA), *Proteus mirabilis*, *Klebsiella pneumonia*, and *Klebsiella oxycata*.

Before experimental use, *Lpb. plantarum* O7S1 was activated in Man Rogosa Sharp (MRS) (Biokar Diagnostics, Allonne, France), while pathogenic bacteria were cultured in nutrient broth (Biokar Diagnostics, Beauvais, France) at 37°C for 18 h. The purity of the cultures was confirmed by streaking on MRS agar and nutrient agar plates, respectively.

### Preparation of the postbiotic metabolites Cell free-supernatants

*Lpb. plantarum* O7S1 was inoculated in MRS broth at 37°C for 18–24 h. After the incubation period, the cells were eliminated by centrifugation at 10 000 × g for 10 min at 4°C. The CFS, containing bioactive metabolites released by this strain, was then filtered using a Millipore filter (0.22 μm) to ensure sterility. The CFS was stored at −20°C until experimental use.

### Exopolysaccharide production

EPS production was achieved by inoculating *Lpb. plantarum* O7S1 in MRS medium with a 1% (v/v) inoculum size (10^9^ CFU/ml) and incubating in a static incubator for 24 h at 37°C. After the incubation period, the bacterial suspension was heated at 95°C for 15 min to inactivate enzymes that could degrade the polymer and liberate cell-attached EPS. After cooling, 80% (w/v) trichloracetic acid (TCA) was added to a final concentration of 4% (v/v). Cells and proteins were removed by centrifugation at 12 000 × g for 30 min at 4°C. The clear supernatant was then mixed with a double volume of cold ethanol (96%), stirred vigorously, and kept at 4°C overnight. The precipitated EPS was separated by centrifugation at 15 000 × g for 12 min at 4°C, and the EPS pellet was suspended in distilled water and stored in the freezer for further analysis (Li et al., 2012). Quantification of EPS was determined by the phenol-sulfuric acid method as described by Dubois et al. (1956).

### Antioxidant activity

#### DPPH free radical scavenging activity

The DPPH (2,2-Diphenyl-1-Picryl-hydrazyl) free radical-scavenging capacity of each postbiotic was investigated using the method described by Yan et al. (2020). Briefly, 1 ml of DPPH solution (0.2 mM in absolute ethanol) was mixed with 1 ml of CFS or EPS solution (1 mg/ml). The reaction solution was vigorously shaken and kept in the dark for 30 min. Ascorbic acid (CARLO ERBA, France) was used as the positive control. The assay was carried out in triplicate. The decrease in absorbance at 517 nm was measured using a UV–visible spectrophotometer (SPECORD 50 Plus, Analytik Jena, Germany), and the scavenging ability was calculated using the following formula (1):


$$\text{Scavenging activity [\%]}=\left[1-\frac{{\text{Abs}}_{\text{sample}}-{\text{Abs}}_{\text{blank}}}{{\text{Abs}}_{\text{control}}}\right]\times 100$$ (1)

where Abs_control_ was the absorbance of (MRS broth or distilled water) and DPPH solution; Abs_sample_ was the absorbance in the presence of samples and DPPH solution and Abs_blank_ was the absorbance in the presence of samples and ethanol (without DPPH solution).

### Hydroxyl radical scavenging activity

The hydroxyl radical scavenging activity was measured following the method proposed by Jia et al. (2019). The mixture contained 0.5 ml of phosphate buffer saline (PBS, pH 7.4), 0.5 ml of 1,10-phenanthroline (2.5 mM), 0.5 ml of FeSO_4_ (2.5 mM), and 0.5 ml of H_2_O_2_ (20 mM). Then, 0.5 ml of CFS or EPS solution (1 mg/ml) was added and incubated at 37°C for an hour to determine the postbiotics’ hydroxyl scavenging capacity. Ascorbic acid was used as the standard, and the absorbance value was measured at 536 nm. The experiment was carried out in triplicate, and the scavenging activity of hydroxyl radicals was determined using the following equation (2):


$$\text{Scavenging activity [\%]}=\frac{{\text{Abs}}_{\text{sample}}-{\text{Abs}}_{\text{control}}}{{\text{Abs}}_{\text{blank}}-{\text{Abs}}_{\text{control}}}\times 100$$ (2)

where Abs_sample_ is the absorbance of the Fenton solution reagent with samples, Abs_control_ is the absorbance of the mixture without the samples, and Abs_blank_ is the absorbance of the mixture without samples and H_2_O_2_ (Fenton solution).

### Superoxide anion scavenging activity

The superoxide anion radical scavenging activity was evaluated using the method described by Zhang et al. (2021). Briefly, 0.1 ml of CFS or EPS solution (1 mg/ml) was mixed with 4.5 ml of Tris–HCl buffer (0.05 M, pH 8.2). The mixture was well shaken and left at 25°C for 10 min, then 0.4 ml of freshly prepared pyrogallol (10 mM) was added. The mixture was kept at 25°C for 25 min, and the reaction was stopped by adding 300 μl of concentrated HCl. Ascorbic acid was used as the positive control, and the test was performed in triplicate. The absorbance was measured at 320 nm, and the superoxide scavenging activity was calculated using the following equation (3):


$$\text{Scavenging activity [\%]}=\left[1-\frac{{\text{Abs}}_{\text{sample}}-{\text{Abs}}_{\text{blank}}}{{\text{Abs}}_{\text{control}}}\right]\times 100$$ (3)

where Abs_sample_ is the absorbance of the postbiotics samples and pyrogallol, Abs_blank_ is the absorbance of the postbiotics and without pyrogallol, and Abs_control_ is the absorbance of the solution with pyrogallol and without postbiotics.

### Ferric reducing antioxidant power

Reducing activity was determined according to the method described by Liang et al. (2016). Briefly, 0.5 ml of CFS or EPS solution (1 mg/ml) was mixed with an equal volume of sodium phosphate buffer (0.02 M, pH 7) and 1% (v/v) potassium ferricyanide. The mixture was then incubated in a 50°C water bath for 20 min. To stop the reaction, 0.5 ml of TCA (10%, w/v) was added, and the mixture was subjected to centrifugation at 800 × g for 10 min. The clear upper layer of the supernatant (1.5 ml) was mixed with 0.2 ml of 0.1% (w/v) ferric chloride (FeCl_3_) and kept at room temperature for 10 min. This experiment was performed in triplicate, and the absorbance was measured at 700 nm. Higher absorbance values indicated greater reducing power. Ascorbic acid was used as the positive reference standard.

### Antidiabetic activity

The α-amylase inhibition assay was performed using the dinitrosalicylic acid (DNSA) method as described by Barros et al. (2020). A total of 250 μl of CFS or EPS solution (1 mg/ml) was added to 250 μl of 0.02 M sodium phosphate buffer (pH 6.9) containing α-amylase solution (0.5 mg/ml). This solution was preincubated at 37°C for 10 min, after which 250 μl of 1% (w/v) starch solution (prepared in 0.02 M sodium phosphate buffer, pH 6.9) was added, followed by incubation at 37°C for 10 min.

The reaction was stopped by adding 500 μl of DNSA reagent (1% DNSA and 12% sodium potassium tartrate in 0.4 M NaOH). The tubes were then incubated in boiling water for 5 min and cooled to room temperature. The reaction mixture was diluted with 5 ml of distilled water, and the absorbance was measured at 540 nm. A similar procedure was followed for the standard drug acarbose (GLUCONOVA®, NOVOPHARM). The test was performed in triplicate, and the α-amylase inhibitory activity was calculated as follows (4):


$$\alpha \;-\text{ amylase inhibition }[\%]=\frac{\left[{\text{Abs}}_{\text{control}}-\left({\text{Abs}}_{\text{sample}}-{\text{Abs}}_{\text{blank}}\right)\right]}{{\text{Abs}}_{\text{control}}}\times 100$$ (4)

where Abs_control_ was the absorbance of control (enzyme without sample solution), Abs_sample_ was the absorbance in the presence of enzyme and sample solution, and Abs_blank_ was the absorbance in the presence of sample solution and without enzyme.

### Antibacterial activity

The agar well diffusion method was used to determine the antimicrobial activity of postbiotics obtained from *Lpb. plantarum* O7S1, as described by Reuben et al. (2020) with some modifications. The indicator pathogens, adjusted to an OD_600_ nm of 0.08–0.1, were swabbed onto Muller Hinton Agar medium using a sterile cotton swab. Then, aliquots (100 μl) of each filtered postbiotic, including CFS and EPS (1 mg/ml), were poured into wells. The CFS was neutralized with sufficient amounts of 1 M NaOH to remove the possible antimicrobial effect of the organic acid (pH 6.5) and compared with non-neutralized CFS. The negative control was filled with MRS broth and distilled water. For rapid diffusion, the plates were kept at 4°C for 2 h and then at 37°C for 24 h. The clear zones formed surrounding the wells were measured and represented in diameters (mm).

### Analysis of the carbohydrate fermentation

The API 50 CH (Biomerieux®, France) gallery consists of 50 microtubes for the fermentation study of 49 compounds, with one control, belonging to the family of carbohydrates and derivatives (glycosides, polyalcohols, and uronic acids). The inoculum was prepared according to the manufacturer’s instructions, and fermentation tests were inoculated with bacterial strain suspension in API 50 CHL medium (bromocresol purple as an indicator of pH). The substrates were rehydrated and covered with 50 μl of paraffin oil to create anaerobic conditions inside the microtubes. The strips were then incubated at 37°C for 48 h. After the incubation period, each microtube was observed for a color change. A positive result was confirmed by a color change of the bromocresol purple indicator from purple to yellow, indicating acid production. However, in the case of well 25 (for the esculin hydrolysis test), the color changed from purple to black. No color change indicated a negative result.

### Statistical analysis

Data from each experiment were expressed as mean ± standard deviation (SD). Statistical analysis was performed using one-way Analysis of Variance (ANOVA) with GraphPad Prism version 8.0.2 (263) (Statistical Software, Inc., United States). Significance was considered at *P* < 0.05, *P* < 0.01, and *P* < 0.001.

## Results

Results appear from this study that *Lpb. plantarum* O7S1 can generate various bioactive metabolites during fermentation in MRS broth, namely CFS and EPS. Following the incubation period, the CFS containing bioactive metabolites was obtained through centrifugation of the fermentation medium, then filtered and stored at −20°C. Moreover, the produced EPS was quantified as total carbohydrates, with a yield of 83.65 ± 9.02 mg/l after 24 h of fermentation. Both produced postbiotics underwent assessment for their biofunctional activities, including antioxidant, antidiabetic, and antibacterial properties.

### Antioxidant activity

**Fig. 1:**
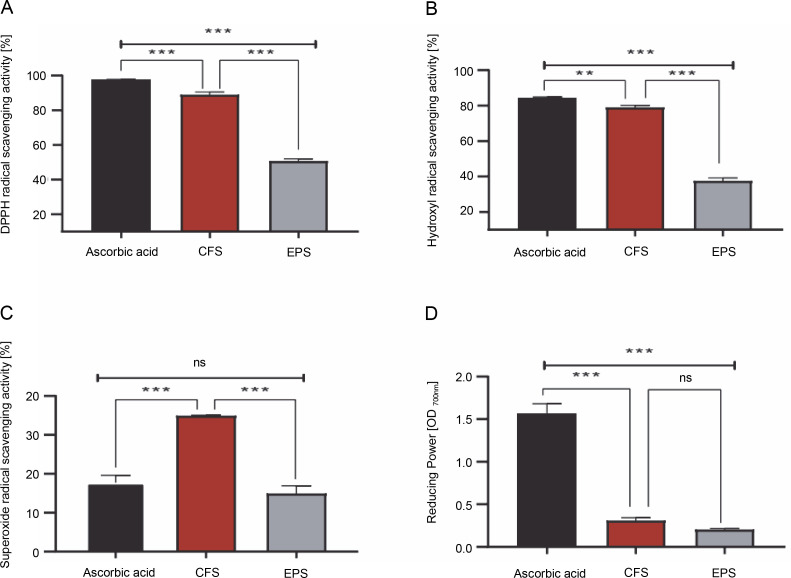
1-diphenyl-2-picrylhydrazyl (DPPH) free radical scavenging activity [%](A), hydroxyl radical scavenging activity [%] (B), superoxide radical scavenging activity [%](C), reducing power OD 700 nm (D) by CFS and EPS of *Lpb. plantarum* O7S1; results are expressed as mean ± SD, (*n* = 3); **P < 0.01, ***P < 0.001 means a significant difference and ns (no significant difference) compared between samples

As shown in Fig. 1A–D, the CFS demonstrated excellent radical scavenging activity and reducing power compared to the EPS (*P* < 0.001). Specifically, the CFS exhibited a remarkable ability to neutralize DPPH, hydroxyl, and superoxide radicals, with inhibition rates of 88.78, 78.91, and 34.85%, respectively. Conversely, EPS displayed comparatively lower inhibition rates against these radicals, with approximately 50.55, 37.40, and 14.86% inhibition towards DPPH, hydroxyl, and superoxide radicals, respectively. Moreover, it was found that the reducing activity of CFS and EPS was 0.30 and 0.20 optical density units (at 700 nm), respectively. On the other hand, the radical scavenging activity and reducing the power of the standard drug, ascorbic acid, were higher than that of our samples (except superoxide scavenging activity) (*P* < 0.001).

### Antidiabetic activity

**Fig. 2:**
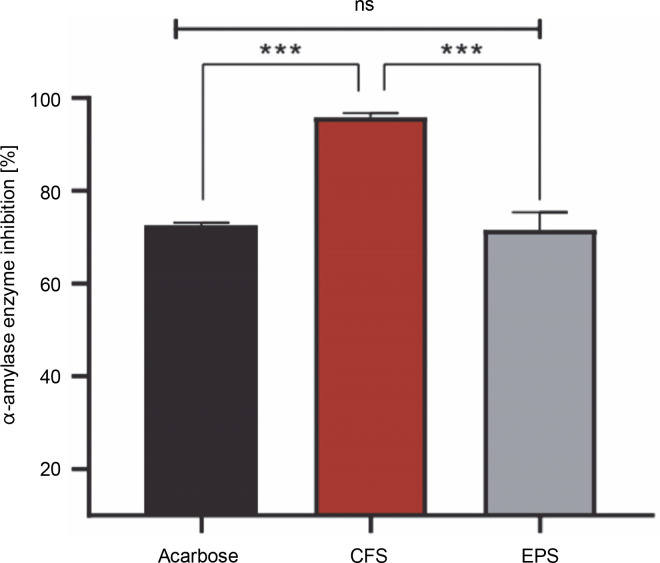
Inhibitory effect against α-amylase by CFS and EPS of *Lpb. plantarum* O7S1; results are expressed as mean ± SD, (*n* = 3); ***P < 0.001 means a significant difference and ns (no significant difference) compared between samples

The percentage of α-amylase inhibition of both postbiotics and acarbose is shown in Figure 2. It was found that the CFS showed a stronger effect with a significant inhibition rate of 95.87%, compared to EPS (71.57%) (*P* < 0.001). However, the difference in inhibition of α-amylase activity between acarbose (72.51%) and EPS was statistically insignificant (*P* > 0.05), but statistically significant with CFS (*P* < 0.001).

### Antibacterial activity

The inhibitory activities of postbiotic metabolites produced by *Lpb. plantarum* O7S1 against Gram-positive and Gram-negative pathogenic bacteria are presented in Table 1. The native CFS (pH 4.2) showed good antagonistic activity against all the tested pathogens. Statistical analysis revealed a significant degree of inhibition of standard indicator strains by CFS (*P* < 0.05), indicating pathogen-dependent inhibition. The largest diameter zones of 18 and 18.5 mm were recorded against *P. aeruginosa* ATCC 27853 and *P. mirabilis*, respectively. However, the lowest inhibition was detected against *E. coli* ATCC 29522 and *S. aureus* ATCC 29523, with zone diameters of 8.5 ± 0.71 and 10.5 ± 0.71 mm, respectively. When the effect of the organic acids was removed by adjusting the pH of CFS to 6.5, no inhibitory effect was detected against any pathogens. Additionally, the EPS (1 mg/ml) from *Lpb. plantarum* O7S1 was unable to inhibit the growth of all the indicator pathogens.

**Table 1:** Inhibition zones diameter (mm) of postbiotics produced by *Lpb. plantarum* O7S1

	*E. coli* ATCC 29522	*P. aeruginosa* ATCC 27853	*P. mirabilis*	*K. pneumoniae*	*K. oxycata*	*S. aureus* ATCC 29523	MRSA
Native CFS	8.5 ± 0.71 ^c^	18± 1.4 ^a^	18.5 ± 0.71 ^a^	14.5 ± 0.71 ^b^	17.5 ± 0.71 ^a^	10.5 ± 0.71 ^c^	17.5 ± 0.71 ^a^
Adjusted CFS	ND	ND	ND	ND	ND	ND	ND
EPS [1 mg/ml]	ND	ND	ND	ND	ND	ND	ND

ND – not detected; cells labeled with the same letters present no significant difference (*P* > 0.05)

### Carbohydrate fermentation

The capacity of *Lpb. plantarum* O7S1 to process 49 types of carbohydrates was assessed using API 50 CH strips. Similar to other major strains of *Lpb. plantarum*, this strain exhibited the ability to metabolize monosaccharides such as glucose, fructose, galactose, mannose, arabinose, ribose, and xylose. Notably, *Lpb. plantarum* O7S1 displayed the capability to degrade disaccharides like melibiose, cellobiose, lactose, trehalose, sucrose, gentiobiose, and turanose, as well as trisaccharides such as melezitose and raffinose. Interestingly, this strain could metabolize esculin, salicin, arbutin, amygdalin, and N-acetylglucosamine, while polysaccharides such as inulin, starch, and glycogen remained indigestible. Additionally, *Lpb. plantarum* O7S1 demonstrated the ability to process polyols, which are alcohols containing three or more hydroxyl groups, including mannitol, sorbitol, and potassium gluconate (Table 2).

**Table 2:** Carbohydrate fermentation profiling for *Lpb. plantarum* O7S1 using the API 50 CH based on 49 different fermentable carbohydrates

Group of species	Reaction
**Monosaccharides**	
L-Arabinose, D-Ribose, D-Xylose, D-Galactose, D-Glucose, D-Fructose, D-Mannose	+
Methyl-αD-Glucopyranoside	–
D-Arabinose, L-Xylose, D-Lyxose, D-Tagatose, D-Fucose, L-Fucose, L-Sorbose, L-Rhamnose	–
Methyl-βD-Xylopyranoside	–
Methyl-αD-Mannopyranoside	–
**Disaccharides**	
D-Celiobiose, D-Maltose, D-Lactose, D-Melibiose, D-Trehalose, D-Sucrose, Gentiobiose, D-Turanose, D-Galactose	+
**Trisaccharides**	
D-Melezitose, D-Raffinose	+
**Polysaccharides**	
Inulin, Starch, Glycogen	–
**Glycerol compounds**	
Esculin, Arbutin, Salicin, Amygdalin, N-Acetylglucosamine	+
**Polyols**	
Glycerol	+/–
D-Sorbitol, D-Mannitol	+
Potassium Gluconate	+
Dulcitol, D-Adonitol, Erythritol, Inositol	–
D-Arabitol, L-Arabitol	–
Potassium 2-Cetogluconate	–
Potassium 5-Cetogluconate	–

(+) – positive reaction, (–) – negative reaction, (+/–) – weakly positive

## Discussion

There is a growing interest in the potential health benefits of probiotic-derived metabolites, often referred to as postbiotics. These compounds have been the subject of extensive research due to their potential positive effects on human health.

The present study aimed to assess the antioxidant activity of both postbiotics, CFS and EPS, at a single dose of 1 mg/ml using different radical scavenging assays and reducing power activity compared to ascorbic acid. The results showed that CFS had greater and stronger antioxidant activity for DPPH free radical (88.78%), hydroxyl (78.91%), and superoxide radical scavenging activity (34.85%) compared to EPS at a concentration of 1 mg/ml (55.55, 37.40, and 14.86%, respectively). However, similar reducing power was recorded for both tested postbiotics (OD_700_ nm ≈ 0.20–0.30). These results suggest that the CFS and EPS produced by this strain could reduce or delay oxidative stress through their ability to scavenge free radicals and their reducing power. Similar observations have been found in earlier studies (Unban et al., 2021; Kim et al., 2022; Noori et al., 2023).

Additionally, it has been reported by Rajoka et al. (2018) that EPS produced by *Lactobacilli* strains display antioxidant activity via free radical scavenging ability and reducing power, which is consistent with the results of this study. It may be hypothesized that the radical scavenging activity of CFS is due to the release of effective antioxidant metabolites by this strain after fermentation, in which these compounds can exhibit potent antioxidant efficacy synergistically or by interacting with other compounds. This assumption was confirmed by Han et al. (2017) and Chen et al. (2015), who indicated that the production of soluble antioxidant compounds, including enzymatic and non-enzymatic antioxidants, proteins, peptides, fatty acids, and polysaccharides in CFS, contributes to the higher antioxidant activity of LAB. On the other hand, the free radical scavenging potential of EPS might be due to the presence of certain hydrogen donor substances, such as hydroxyl and carboxyl groups, which can react with free radicals and convert them into more stable molecules (Zhang et al., 2021).

It is worth noting that diabetes is a chronic metabolic disorder characterized by high blood sugar concentrations, which can lead to serious health complications (Chen et al., 2014). Therefore, reducing blood glucose levels is the best way to prevent and control diabetes. Barros et al. (2020) reported that inhibiting the activity of carbohydrate-hydrolyzing enzymes, such as α-amylase and α-glucosidase, is one of the possible mechanisms for controlling diabetes.

In this study, we explored the antidiabetic properties of our postbiotics through their inhibitory effects on α-amylase activity. Although we used acarbose, an oral hypoglycemic drug, as a positive control, it is important to note that acarbose’s action alone may not encompass the full spectrum of natural compounds or postbiotics derived from LAB, which could exhibit diverse mechanisms of action in the management of diabetes. However, this standard drug is renowned for its capacity to inhibit both α-amylase and α-glucosidase enzymes, which are among the potential mechanisms for managing diabetes.

The obtained results revealed that both EPS and CFS had potent α-amylase inhibitory activity (67.17 and 95.87%, respectively), which was comparable to or higher than 1 mg/ml of acarbose (72.51%). These results are consistent with numerous reports, such as the study by Won et al. (2021), which showed that α-amylase inhibitory activities of culture supernatant of 17 strains of LAB ranged from 57 to 88.7%. In a relevant study, Kim et al. (2018) reported that *Lpb. plantarum* K10 showed potent inhibition of α-amylase activity by 94.6%. Consistent with our results, Dilna et al. (2015) and Ayassh et al. (2020) demonstrated that EPS produced by LAB strains effectively inhibited α-amylase.

The obtained results clearly showed that CFS and EPS from *Lpb. plantarum* O7S1 has hypoglycemic activity by inhibiting the carbohydrate-metabolizing enzyme α-amylase. While the precise mechanisms of α-amylase activity inhibition by postbiotics are yet unknown, it is speculated that the postbiotics contain some α-amylase inhibiting compounds or that the postbiotics compete with starch by binding the active sites of α-amylase, making the substrate unavailable for hydrolysis by the enzyme.

In addition to their antioxidant and antidiabetic properties, postbiotics derived from LAB have attracted much attention due to their antibacterial effects against pathogenic and food spoilage bacteria (Aghebati-Maleki et al., 2021). This ability to inhibit pathogens provides a sustainable alternative to antibiotics. One of the most important postbiotics known for its antimicrobial activity is CFS. It is considered a consortium of diverse fermentative metabolites with bacteriostatic or bactericidal activities, including low molecular weight compounds such as organic acids, di-acetylene, carbon dioxide, and reuterin, as well as high molecular weight substances like polysaccharides and bacteriocins (Nataraj et al., 2020).

In this study, the antagonistic effect of postbiotics (CFS and EPS) derived from *Lpb. plantarum* O7S1 was investigated using the agar well diffusion method. Our results showed that the CFS (pH 4.2; after fermentation in MRS broth) exhibited good antibacterial activity with a significant and varying degree of inhibition against Gram-negative and Gram-positive pathogens. This aligns with the study by Rastogi et al. (2020), who confirmed that the CFS of *Lactobacillus mucosae* and *Lactobacillus gasseri* displayed a significant strain-specific nature.

It is very important to determine the metabolite responsible for the antimicrobial effects exerted by CFS. In this study, when the pH value of the CFS was adjusted to 6.5 with NaOH, no inhibition zones were detected around the wells of all the tested pathogens, indicating that the antagonistic activity of CFS is indeed pH-dependent. The absence of pathogen inhibition in pH-neutralized CFS confirms that the metabolite-causing antimicrobial activity results from the production of acidic metabolites (such as organic acids like lactic acid) rather than bacteriocins and other metabolites.

Our results concur with those of Khalil et al. (2018) and Unban et al. (2021), who demonstrated that organic acid production is the main antimicrobial strategy of LAB, especially *Lactiplantibacillus* sp. It is important to note that organic acids disrupt the integrity of the cell membrane, leading to the leakage of cell contents. This disruption is one of the mechanisms through which organic acids can exert their antimicrobial effects (Aghebati-Maleki et al., 2021).

Moreover, our results indicate that EPS of *Lpb. plantarum* O7S1 (1 mg/ml) had no antibacterial activity against all pathogens. This result contradicts the findings of Rajoka et al. (2018), who reported that EPS produced by *L. reuteri* SHA101 and *L. vaginalis* SHA110 showed antibacterial activity against both Gram-positive and Gram-negative bacteria. This contradiction may be attributed to the type of probiotic from which the postbiotic is prepared, as the beneficial effects of postbiotics depend exclusively on the type of probiotic and the medium used to extract the postbiotics (Aghebati-Maleki et al., 2021). Several studies have reported that the production and functionality of postbiotics are strain-dependent (Aydin et al., 2021; Diez-Gutiérrez et al., 2022).

As reported previously, LAB postbiotics are metabolites produced by cells during the fermentation process in culture media, food, or the intestinal gut (Aghebati-Maleki et al., 2021; Wang et al., 2021). Carbohydrates are one of the essential compounds present in food products and, therefore, in the human diet. Various researches have shown that carbohydrates resistant to digestion and those that escape absorption in the small intestine are available for gut fermentation, resulting in the production of various postbiotic compounds inside the gastrointestinal tract (Wong and Jenkins, 2007; Wang et al., 2021; Diez-Gutiérrez et al., 2022).

In this case, incorporating probiotic strains with carbohydrate fermentation capabilities into one’s diet or supplement regimen can help modulate the gut microbiota and promote overall gut health. However, it is essential to choose probiotic strains carefully; as the specific effects may vary depending on the strain and the types of carbohydrates they can ferment.

In this regard, the ability of *Lpb. plantarum* O7S1 to process 49 types of carbohydrates was evaluated using API 50 CH strips. Overall, it was found that this strain could metabolize different monosaccharides, including galactose, fructose, glucose, ribose, mannose, and arabinose. Generally, these sugars are easily used as a source of carbon and energy to enhance the growth of gut microbiota. Disaccharides and trisaccharides such as melibiose, cellobiose, lactose, trehalose, sucrose, gentiobiose, turanose, melezitose, and raffinose were also metabolized by this strain. Gebreselassie et al. (2016) reported that *Lpb. pentosus* isolated from Ethiopian naturally fermented buttermilk could catabolize all these carbohydrates, except melezitose. Mao et al. (2018) showed that raffinose catabolism can stimulate the growth of probiotic microorganisms, maintain gut functionality, and increase the absorption of minerals such as zinc, calcium, magnesium, and iron, which has been proven in animal and human models (Rafique et al., 2023).

Additionally, this lactic acid bacterium can transform mannitol, sorbitol, and potassium gluconate. Degradation of these sweeteners by LABs can improve the digestion process and stimulate the synthesis of two postbiotics (lactic and butyric acid), preserve a healthy intestine, and increase the absorption of some nutrients (Diez-Gutiérrez et al., 2022). Interestingly, this strain highlighted the ability to catabolize amygdalin, which is not always found in all *Lpb. plantarum* strains. Consequently, the ability to metabolize amygdalin could be regarded as a valuable probiotic trait, as this sugar (a phytotoxic secondary metabolite) has the potential to pose cytotoxic effects on human health. This highlights its importance in enhancing dietary tolerance and promoting overall health. These findings are confirmed by other studies such as those by Diez-Gutiérrez et al. (2022) and Iorizzo et al. (2022).

Overall, the obtained data highlighted that both postbiotics, particularly the CFS of *Lpb. plantarum* O7S1, exhibits high antioxidant activity compared to EPS concerning their DPPH, hydroxyl, and superoxide radical scavenging activity, as well as their reducing power. Furthermore, CFS showed a significant percentage of inhibition of α-amylase activity, suggesting its antidiabetic property. Similarly, native CFS also demonstrated potent antagonistic activity toward Gram-positive and Gram-negative pathogens, indicating its antibacterial activity. The relatively low biological effect observed for EPS in this study may be attributed to the concentration utilized, as previous studies have indicated the dose-dependent nature of EPS therapeutic effects (Dilna et al., 2015; Jia et al., 2019; Zhang et al., 2021). To avoid this limitation, future research will explore different concentrations of postbiotics and aim to identify the specific bioactive compounds. Furthermore, additional research focusing on animal models to validate the bioactivities of both postbiotics is recommended.

## Conclusion

An important finding to emerge from this study is the ability of the novel *Lpb. plantarum* O7S1, isolated from fermented olives, to produce two biofunctional postbiotics (CFS and EPS) in MRS broth, with CFS being more effective than EPS in terms of antioxidant, antibacterial, and antidiabetic activities. In addition, this lactic acid bacterium has demonstrated its ability to promote the digestion of carbohydrates and their derivatives. This could be beneficial for individuals with digestive issues or those looking to improve nutrient absorption. These encouraging findings suggest a promising application for our postbiotics, particularly CFS, as alternative agents in both the food and pharmaceutical industries for mitigating oxidative stress, diabetes, and combating pathogenic bacteria. Nevertheless, future research, particularly focusing on *in vivo* studies, is recommended to further explore this topic.

## Data Availability

Data and material are contained within the article.
